# Rosy Discolouration in an Alpine Chapel: Beyond Salt Dependence

**DOI:** 10.1007/s00248-026-02795-2

**Published:** 2026-05-27

**Authors:** Alessia Marzanni, Maria Landolfi, Raphael Tiziani, Sabrina Bombardelli, Domenico Celi, Martin Pittertschatscher, Alessia Buttarelli, Silvia Bruni, Elena Pecchioni, Brunella Perito, Veerle Cnudde, Francesca Cappitelli, Tanja Mimmo, Federica Villa, Luigimaria Borruso

**Affiliations:** 1 Faculty of Agricultural, Environmental and Food Sciences, Free University of Bozen- Bolzano, Bolzano, 39100 Italy; 2 PProGRess-UGCT, Department of Geology, Ghent University, Ghent, Belgium; 3 Competence Centre for Plant Health, Free University of Bozen-Bolzano, Bolzano, Italy; 4 Department of Biology, Università degli Studi di Firenze, Florence, Italy; 5 Associazione Restauratori-Conservatori Alto Adige (ARCA), Bolzano, Italy; 6 Department of Chemistry, Università degli Studi di Milano, Milan, Italy; 7 Department of Earth Sciences, Università degli Studi di Firenze, Florence, Italy; 8 Environmental Hydrogeology, Department of Earth Sciences, Utrecht University, Utrecht, the Netherlands; 9 Department of Food, Environmental and Nutritional Sciences, Università degli Studi di Milano, Milan, Italy

**Keywords:** Biofilm pigmentation, Rosy discolouration, Mountain cultural heritage, Microbiome, Surface chemistry, Salt efflorescence

## Abstract

Stone heritage is central to cultural identity, yet alpine monuments remain understudied and vulnerable to deterioration. In mountain environments, moisture and temperature fluctuations promote salt crystallisation in porous geomaterials, frequently associated with rosy discolouration attributed to pink biofilms, a phenomenon still poorly explored at higher elevations. Here, we investigated rosy discolouration at about 1000 m altitude on the walls of St. Cyprian Chapel (South Tyrol) using metabarcoding and chemical analyses. We compared two cases of rosy discolouration: one on the north wall, where salt efflorescence is present, and one on the south wall, where it is absent. The aim was to characterise how salt efflorescence modulates rosy discolouration by comparing the two walls and linking substrate chemistry and mineralogy to biofilm pigments, structure, and microbial community composition. Despite similar mineralogy, the north wall exhibited distinct surface chemistry consistent with salt efflorescence. Biofilm biomass was comparable between walls, and no photosynthetic organisms were detected in either biofilm, yet microbial communities differed markedly. The south wall hosted carotenoid-producing biomarkers, including *Rubrobacter* and *Pontibacter*, although Raman did not detect carotenoid signals, indicating low pigment expression. In contrast, the north wall showed stronger discolouration and clear detection of bacterioruberin. Notably, the north-wall biomarker *Chryseobacterium* may also contribute to the rosy discolouration through flexirubin-type pigments. Overall, salt efflorescence is not required for pink biofilm establishment, but salt-rich microhabitats appear to enhance pigment production. This study provides new insights into the drivers of rosy discolouration and contributes to the still-limited research on alpine cultural heritage.

**Supplementary Information:**

The online version contains supplementary material available at 10.1007/s00248-026-02795-2.

## Introduction

Stone heritage is deeply linked to our historical and cultural legacy, making its conservation a shared responsibility for both present and future generations. In mountain areas, stone heritage is more than a legacy from the past; it is a key resource for sustainable development, resilience, and vitality of local inhabitants [1]. By strengthening socio-economic development and reinforcing cultural identity, stone heritage plays a crucial role in supporting the well-being of mountain communities and helping counter depopulation.

The close interaction between stone heritage and nature in mountain areas makes monuments especially vulnerable to ongoing environmental changes, which can accelerate their weathering [2, 3]. In particular, frequent fluctuations in moisture and temperature promote crystallisation of salt on the surface, contributing significantly to material deterioration [4]. Indeed, in porous construction materials, hygroscopic salts can dissolve and migrate within the pore structure in response to environmental changes, leading to crystallisation-dissolution cycles that generate pressure, detach plaster, form cracks, and produce visible salt efflorescence on surfaces [5, 6].

The presence of soluble salts also appears to influence microbial surface colonisation. The repeated co-occurrence of salt efflorescence and rosy discolouration has been widely documented in historical buildings worldwide and is frequently associated with the development of pinkish biofilms [7, 8]. The distinctive colouration of these pinkish biofilms is attributed to the production of carotenoid-like pigments, in particular bacterioruberin, which act as membrane stabilisers against salt stress and desiccation [9, 10]. Previous studies have shown that salt-weathered buildings across different geographical locations share a core microbiome, composed mainly of halotolerant and halophilic bacteria [11], algae [12], and archaea [7].

**Fig. 1:**
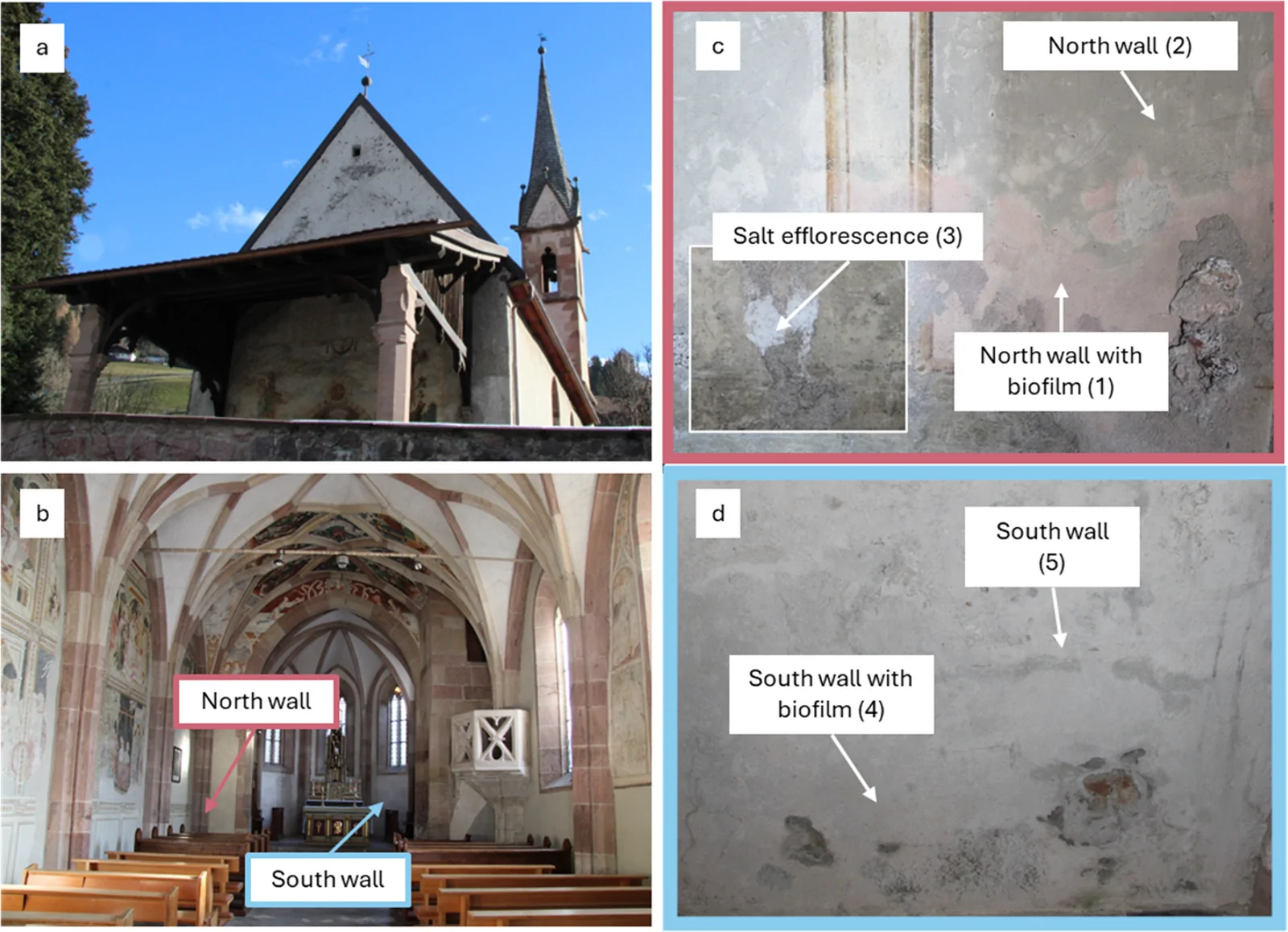
Overview of the sampling areas inside the studied church. The exterior of the building (**a**) and the interior view showing the positions of the north and south walls (**b**). The north wall (**c**), indicating the sampled areas: surface with biofilm (1), surface without biofilm (2), and a zone affected by salt efflorescence (3). The south wall (**d**), showing the sampled areas: surface with biofilm (4) and adjacent surface without biofilm (5)

Despite over two decades of research on pink biofilms associated with salt-weathered surfaces, rosy discolouration in mountain environments remains relatively understudied, with limited attention so far given to alpine monuments at elevations of approximately 1000 m and above. Here, we address this gap by studying rosy discolouration on the north and south walls of St. Cyprian’s Chapel (Sarentino, South Tyrol, Italy; ~1000 m a.s.l.), where efflorescence occurs only on the north wall. First documented in 1328, it dates to the 14th century and was renovated in the 15th century, resulting in its current Gothic appearance. The chapel stands near a terrace protected by a low wall (Fig. 1a) and consists of a single nave containing an important fresco cycle attributed to a Giottesque school, dated to the 14th -15th centuries (Fig. 1b). The interior walls show contrasting deterioration patterns: on the north wall, plaster is peeling or missing, with salt efflorescence near patches of rosy discolouration (Fig. 1c), while the south wall shows minor deterioration, with only light rosy discolouration and no visible signs of salt efflorescence (Fig. 1d). Thus, this study aims to characterise how salt efflorescence modulates rosy discolouration by comparing a salt-affected north wall with a south wall without salt efflorescence, and by linking substrate chemistry and mineralogy to biofilm pigments, structure, and microbial community composition. We hypothesise that salt efflorescence enhances pigment production, leading to more intense rosy discolouration. However, we propose that salt is not a strict prerequisite for this phenomenon, which may also occur under non-saline conditions, but likely with lower pigmentation intensity.

## Materials and Methods

### Sampling Design

This research focused on the north and south walls of St. Cyprian’s Chapel (Fig. 1), specifically the lower portion of the fourth arch on the north wall and the apse area on the south wall, both selected for their visible rosy discolouration. Replicates were collected when feasible, although sometimes limited by sample availability and conservation constraints (Table S1). For chemical analyses, four replicates were taken from biofilm-covered areas and two from biofilm-free areas on each wall, plus one salt efflorescence sample from the north wall. Samples for chemical analyses were obtained by scraping the surface material and stored at room temperature in Falcon tubes or plastic bags. For XRD, three additional replicates were collected from biofilm-covered surfaces of each wall, and three salt efflorescence replicates from the north wall, using the same sampling and storage procedure as for chemical analyses. Raman spectroscopy and micro-spectrofluorimetry were conducted on one biofilm-covered and one biofilm-free sample per wall; small flakes of the substrate were collected for these analyses. For confocal laser scanning microscopy (CLSM) analyses, one sample was collected from each biofilm using adhesive fungi tape and stored at room temperature; five measurements were performed at different points on each sample. For metabarcoding analysis, four biological replicates were collected from each rosy discolouration (north and south). Microbiological samples were collected from both walls using a sterile scalpel and stored in sterile Falcon tubes at − 20 °C. Colourimetric analyses were performed in situ, with five measurements per area (north wall with biofilm, north wall without biofilm, south wall with biofilm, south wall without biofilm). All samples or measurements were collected 20–30 cm apart within ~ 1 m² per wall.

### Chemical and Colourimetric Analyses

Samples were homogenised using a Retsch mixer mill MM400 (Retsch, Germany) with agate balls prior to analysis. For the pseudo-total elemental content, ~ 0.1 g of each sample was acid-digested with concentrated ultrapure HNO_3_ (69% w/v) using a single reaction chamber microwave digestion system (UltraWAVE, Milestone, Shelton, CT, United States). The samples were then diluted to 10 mL with laboratory-grade I water, filtered through a 0.45 μm cellulose Whatman filter (grade 42). The digestate was then analysed by Inductively Coupled Plasma Mass Spectroscopy (ICP-MS, Agilent 7800, United States) [13], using lanthanum as an internal standard at 1 ppm and normalising the results by sample weight. The total carbon (C) and nitrogen (N) content was determined using the Flash EA 112 Elemental Analyser (Thermo Scientific, Germany) after drying samples overnight at 70 °C and weighing 5 mg into tin capsules. For pH, 250 mg of each sample was mixed with laboratory-grade I water (1:2.5 sample-to-water ratio), vortexed for 10 min and centrifuged at 4,000 × g for 10 min at room temperature to separate the supernatant for pH measurement. A calibrated portable pH meter (pH7 Vio, Metrocal, Italy) with a microelectrode was then inserted into the supernatant to measure the pH.

Biofilm pigments were characterised by Raman spectroscopy and micro-spectrofluorimetry. Raman analyses were performed using a Jasco RMP100 micro-probe equipped with a notch filter and an Olympus 50X objective. The probe is connected via optical fibre to a Lot-Oriel MS 125 spectrometer with a 1800 lines/mm grating and a CCD Andor detector (1024 × 128 pixels) cooled by a Peltier device. The probe is also connected to a frequency-doubled ND-YAG laser source with emission at 532 nm (green). The spectra were acquired as a sum of 30 scans with an exposure time of 2 s between 300 and 2700 cm^− 1^. Micro-spectrofluorimetric analyses were performed using the same instrumental setup described above, with a 400 lines/mm grating. Spectra were acquired as the sum of 30 scans with an exposure time of 0.01 s over the spectral region 500–900 nm. All the analyses were preceded by the acquisition of a background spectrum in dark conditions.

Chromatic parameters were measured with a Chroma Meter CR-400/410 colourimeter (Konica Minolta, Tokyo, Japan) in the CIE L*a*b* colour space. Colour differences (∆E) were calculated according to Eq. 1, where L*, a*, and b* represent the coordinates in the CIELAB colour space. Three types of comparisons were made: (i) north vs. south walls with biofilm; (ii) north wall with vs. without biofilm; (iii) south wall with vs. without biofilm.

1 $$\triangle E\;=\;\sqrt\triangle\;\left(L\ast\right)^2\;+\;\triangle\;\left(a\ast\right)^2\;+\;\triangle\;\left(b\ast\right)^2$$

### Mineralogical Analysis

X-ray diffraction (XRD) analysis was performed using a Philips PW 1050/37 diffractometer, equipped with an X’Pert PRO data acquisition system and processed with HighScore software. The instrument operated at 40 kV and 20 mA, using a Cu anode and a graphite monochromator. Data were collected over a 2θ range of 5–70°, with a detection limit of 4%. The results of the qualitative mineralogical analyses were expressed as partial estimates of the mineral amounts based only on peak elongation.

### Confocal Laser Scanning Microscopy (CLSM)

Biofilm samples were stained with the fluorescent nucleic acid stain SYTO9 (Thermo Fisher Scientific, Italy) according to the manufacturer’s instructions. Confocal images were acquired using a Nikon A1/A1R confocal microscope (Nikon Instruments Inc., Amstelveen, Netherlands) equipped with a 20x dry objective. Fluorescence was excited and detected using the following settings: SYTO9 (total biomass), excitation at 488 nm and emission at 500–550 nm; autofluorescence of photosynthetic microorganisms, excitation at 633 nm and emission at 650–750 nm. Reflectance-mode imaging was performed using the 488 nm argon laser line to obtain surface-relief images. Three-dimensional reconstructions were generated using ImarisViewer (Bitplane Scientific Software, Switzerland). Biovolumes were calculated using ImageJ.

### DNA Extraction, Library Preparation, and Bioinformatics Analysis

Before extraction, samples were ground in a sterile mortar. DNA was extracted using the DNeasy PowerSoil Pro kit (Qiagen) following the manufacturer’s protocol, quantified with a Qubit 4 Fluorometer (Thermo Fisher Scientific), and, due to low yields, concentrated using the DNA Clean & Concentrator^®^-5 kit (ZYMO RESEARCH). The DNA was then sent to AllGenetics (A Coruña, Spain) for library preparation and sequencing. During library preparation, the V3–V4 region of the bacterial 16 S rRNA gene was amplified with primers 341 F and 805R [14], while the archaeal 16 S rRNA (V3–V4) was amplified with primers 340 F/806R [15]. For fungi, the ITS2 region was amplified using primers ITS3-ITS4 [16]. Sequencing was performed on an Illumina NovaSeq platform using pairedend 2 × 250 bp reads (PE250).

Raw sequencing data for each dataset (bacterial 16 S rRNA gene, archaeal 16 S rRNA gene, and fungal ITS2 region) were independently pre-processed, quality-filtered, trimmed, denoised, merged, modelled, and analysed using QIIME2 [17] and DADA2 [18]. Chimaeras were checked and removed using the ‘consensus method’ with DADA2 [18], and subsequently amplicon sequence variants (ASV) were clustered in Operational Taxonomic Units (OTUs) using VSEARCH within QIIME2, with a 97% similarity threshold [19]. Finally, taxonomy was assigned using UNITE + INSD database (v10.0) for fungal OTUs, and SILVA database (v138.2) for both Bacteria and Archaea [20]. The complete bioinformatic workflow is described in the Supplementary Material and is publicly available in the GitHub repository SoilMolecularEcology/metabarcoding-workflows (subdirectory articles/rosy_discolouration-alpine-chapel).

Sequencing output after quality-control filtering, including total retained reads, average reads per sample, number of OTUs, and number of samples analysed (non-rarefied datasets), is summarised in Supplementary Table S2.

### Statistical Analysis

Data analysis of pH, elemental composition, total C and N, and biofilm biomass was performed in RStudio (version 4.4.1) (Team, 2024). For each variable, mean and standard deviation were calculated, and normality was assessed using the Shapiro–Wilk test. Group differences were tested using one-way ANOVA for normally distributed data or Kruskal–Wallis for non-normal data, followed by Tukey’s HSD (ANOVA) or Dunn’s test with Bonferroni correction (Kruskal–Wallis). Principal Component Analysis (PCA) was used to explore multivariate patterns and relationships among variables. Statistical significance was set at *p* < 0.05.

Metabarcoding data were analysed in RStudio (v4.4.1) (Team, 2024). Beta diversity between microbial communities from the north and south biofilms was assessed using Bray–Curtis dissimilarities, visualised by cluster analysis, and tested using permutational ANOVA (PERMANOVA; 999 permutations). Biomarker taxa associated with each biofilm were identified using LEfSe (Linear Discriminant Analysis Effect Size), applying thresholds of LDA score > 2 and *p* < 0.05.

## Results

### Chemical and Mineralogical Characterisation

**Fig. 2:**
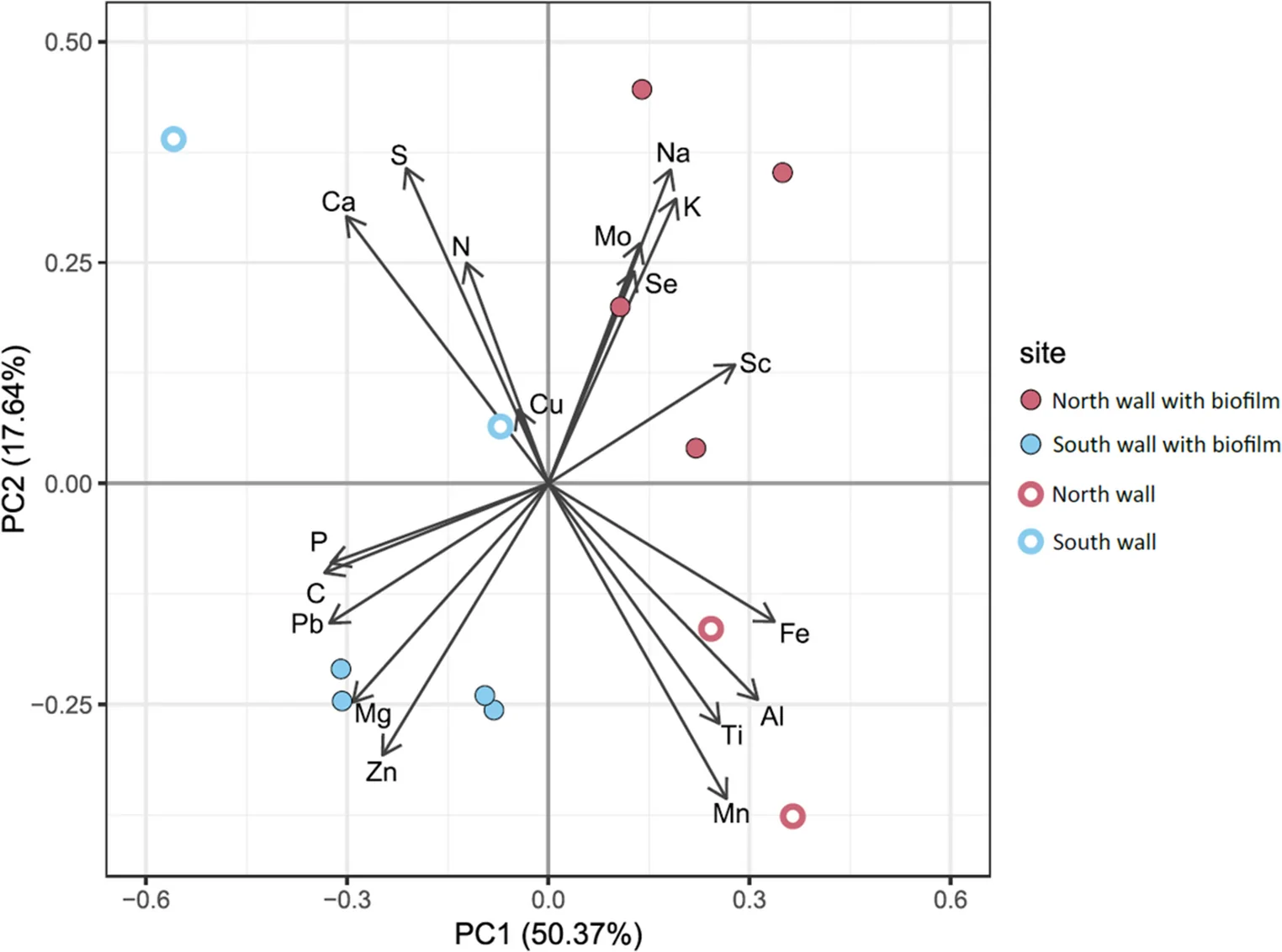
Principal component analysis (PCA) of the north and south walls with and without biofilm, based on elemental composition. Pink symbols correspond to the north wall and light blue symbols to the south wall. Filled symbols represent biofilm-covered samples, while open symbols indicate biofilm-free substrates

The PCA based on chemical composition and pH (Fig. 2) showed a clear separation between north and south wall samples (with and without biofilm), mainly along the first principal component (PC1, 50.37% of variance) (Fig. 2). North wall samples exhibited a higher content of sodium (Na), potassium (K), selenium (Se), molybdenum (Mo), scandium (Sc), iron (Fe), aluminium (Al), manganese (Mn), and titanium (Ti), whereas south wall samples showed higher C, magnesium (Mg), zinc (Zn), phosphorus (P), lead (Pb) and calcium (Ca). The second principal component (PC2, 17.64% variance) (Fig. 2) further separated samples according to biofilm presence, but with opposite trends on the two walls: on the south wall, biofilm samples clustered at negative PC2 values and were associated with Mg and Zn, while biofilm-free samples plotted at positive PC2 values and were linked to sulphur (S) and Ca; on the north wall, biofilm samples plotted at positive PC2 values and were associated with Na and Se, whereas biofilm-free samples were associated with C and P.

Several elements differed significantly among sample types (*p* < 0.05; post-hoc P.adj < 0.05), with most significant differences occurring between the north and south walls rather than between biofilm and no-biofilm areas on the same wall (Table S3). South wall showed higher Mg (7.1-fold higher in south with biofilm vs. north with biofilm, P.adj = 0.036), P (+ 42% south vs. north wall, P.adj = 0.014; +37% south with biofilm vs. north with biofilm, P.adj = 0.010), Ca ( 2.40-fold higher in the south wall vs. the north wall, P.adj = 0.003), and Zn (50-fold higher south wall with biofilm vs. north wall with biofilm, P.adj = 0.010). Total N was also higher in the South (12-fold, + 1100% south vs. north wall, P.adj = 0.009), as was total C (+ 212% south vs. north wall, P.adj = 0.004; +127% south with biofilm vs. north with biofilm, P.adj = 0.0007). Conversely, several elements were more abundant in the North: Na (+ 840% north wall with biofilm vs. south wall, P.adj = 0.023), Al (+ 79% north vs. south wall, P.adj = 0.026), K (+ 502% north wall with biofilm vs. south wall without biofilm, P.adj = 0.023), and Fe (+ 96% north vs. south wall, P.adj = 0.022) (Table S3).

pH was consistently higher in the north (+ 8% south vs. north wall, P.adj = 0.001; +7% south with biofilm vs. north with biofilm, P.adj = 0.0004) and was the only chemical parameter significantly differing within the same wall, with south wall biofilm showing + 4% higher pH than south wall without biofilm (P.adj = 0.036). TC/TN ratios did not differ significantly between groups (Kruskal–Wallis, *p* = 0.052) (Table S3).

Salt efflorescence from the north wall showed a predominance of Na (+ 1732% compared to north wall with biofilm), followed by S, Ca, and K. Pb was not detected, while elements detected in traces (µg/g) include P, Ti, Mn, Cu, Zn, Mo, Sc, and Se. Total N and C were 0.11% and 2.33%, respectively, and pH was strongly alkaline (10.25), matching the north wall value (10.25 ± 0.16) (Table S3).

XRD analysis showed that wall samples (mortars and plasters) consisted of quartz, calcite, plagioclase, K-feldspar, clay minerals, and micas (Table S4), while salt efflorescence was composed of magnesium carbonates (MgCO_3_) and sulphates (MgSO_4_), specifically epsomite, nesquehonite, dypingite, and hydromagnesite (Table S5).

### Pigment and Colourimetric Analysis

**Fig. 3:**
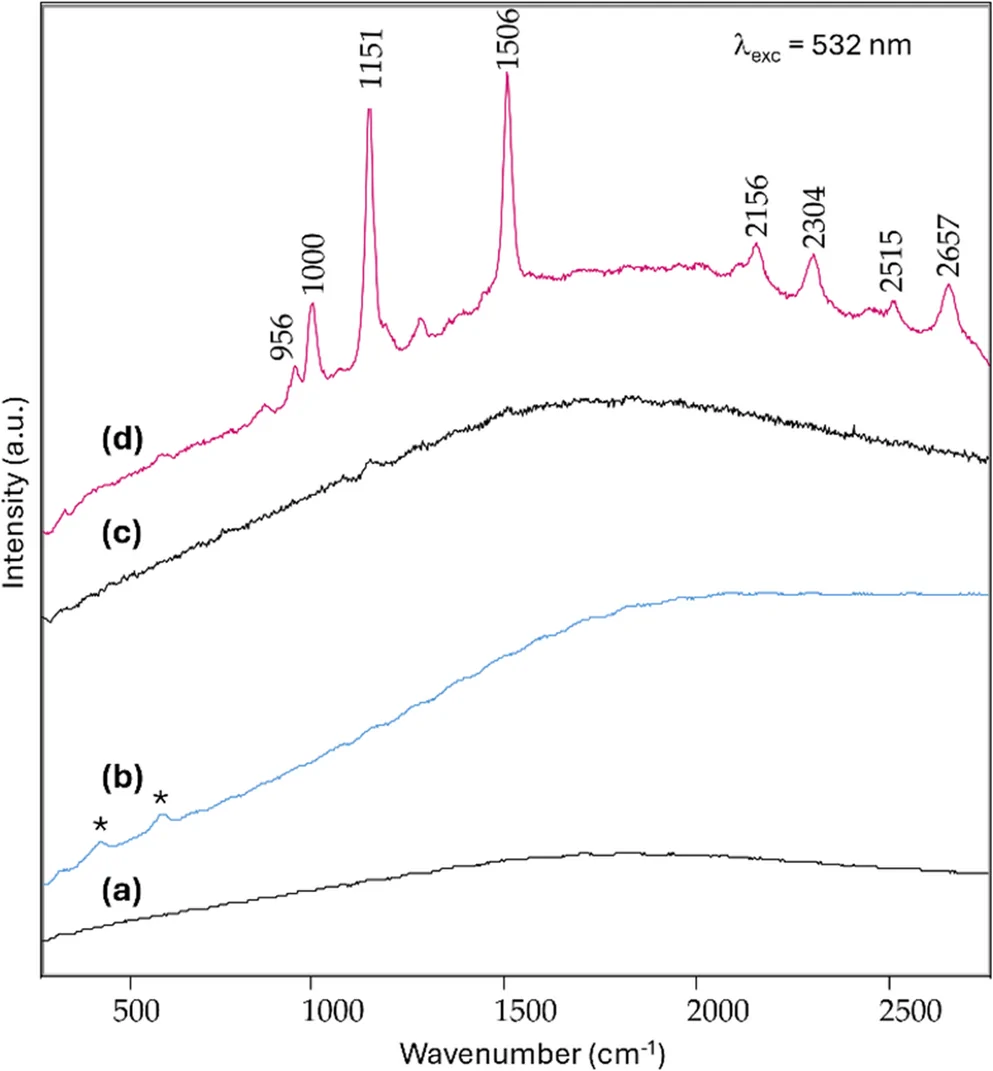
Micro-Raman spectra (λ_exc_ = 532 nm) of the south wall without biofilm (**a**), the south wall with biofilm (**b**), the north wall without biofilm (**c**), and the north wall with biofilm (**d**). The bands marked with an asterisk are due to titanium dioxide TiO_2_ as rutile

The Raman spectrum obtained from the biofilm on the north wall exhibited strong signals between 1500 –1000 cm^− 1^, characteristic of carotenoid pigments (Fig. 3), which were absent in the biofilm-free wall spectrum. Peaks at 1506, 1151, and 1000 cm^− 1^ indicate the presence of bacterioruberin pigment [21]. In the higher wave number region, harmonic combination bands typical of carotenoids were observed at 2657, 2515, 2304, and 2156 cm^− 1^ (Fig. 3) [22], which confirmed carotenoid-related pigments. No detectable Raman signal was obtained from the south wall biofilm, suggesting that pigment concentrations were below the detection limit.

Fluorescence spectra of both biofilms revealed a broad band with an emission maximum around 600 nm (Fig. S1). However, only the north biofilm showed significantly higher intensity than the substrate, with carotenoid Raman signals superimposed. This pattern was consistent with carotenoid emission spectra reported in the literature [23].

**Table 1:** Color difference (ΔE) between the north wall with and without biofilm, the south wall with and without biofilm, and between biofilm-covered areas on the north and south walls

Comparison	ΔE
North biofilm vs. north wall	5.36
South biofilm vs. south wall	4.28
North biofilm vs. south biofilm	8.20

**Fig. 4:**
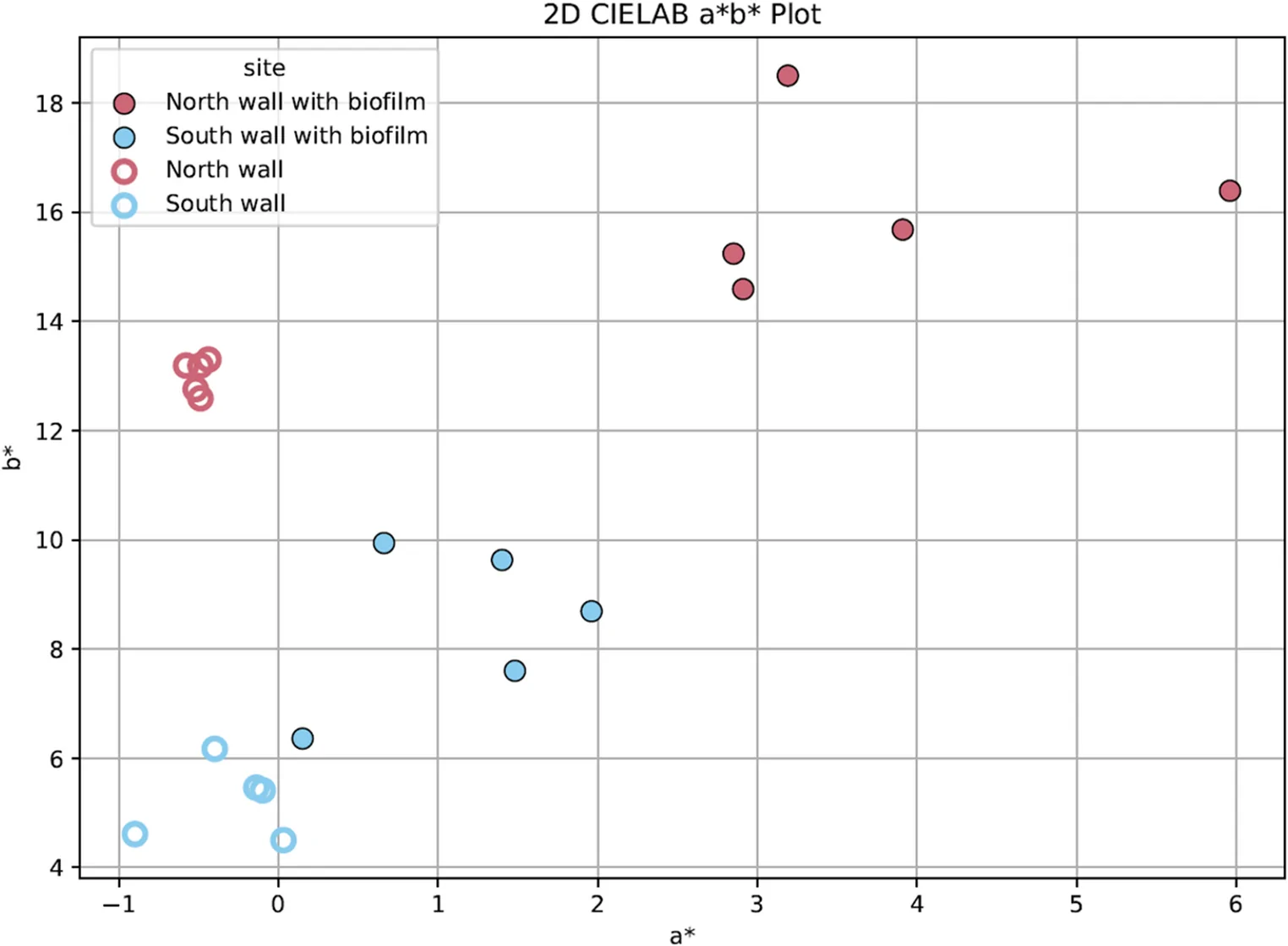
a*b* graph showing the results of the colorimetric analysis of the north wall and north wall with biofilm, and of the south wall and south wall with biofilm

Colourimetric revealed clear differences between areas with and without biofilm on both walls, more pronounced on the north (ΔE = 5.36) than on the south (ΔE = 4.28) (Table 1). The ΔE value for the north wall exceeds the commonly accepted perceptibility threshold (ΔE > 5), above which colour differences are generally considered visible to the human eye, whereas the south wall shows a difference close to this threshold. The a*b* graph showed distinct separation among the four groups (north/south, with/without biofilm) (Fig. 4), with generally lower a* and b* values in south samples. The ΔE between north and south biofilm-covered areas was 8.2.

### Biofilm Imaging and Taxonomic Composition

**Fig. 5:**
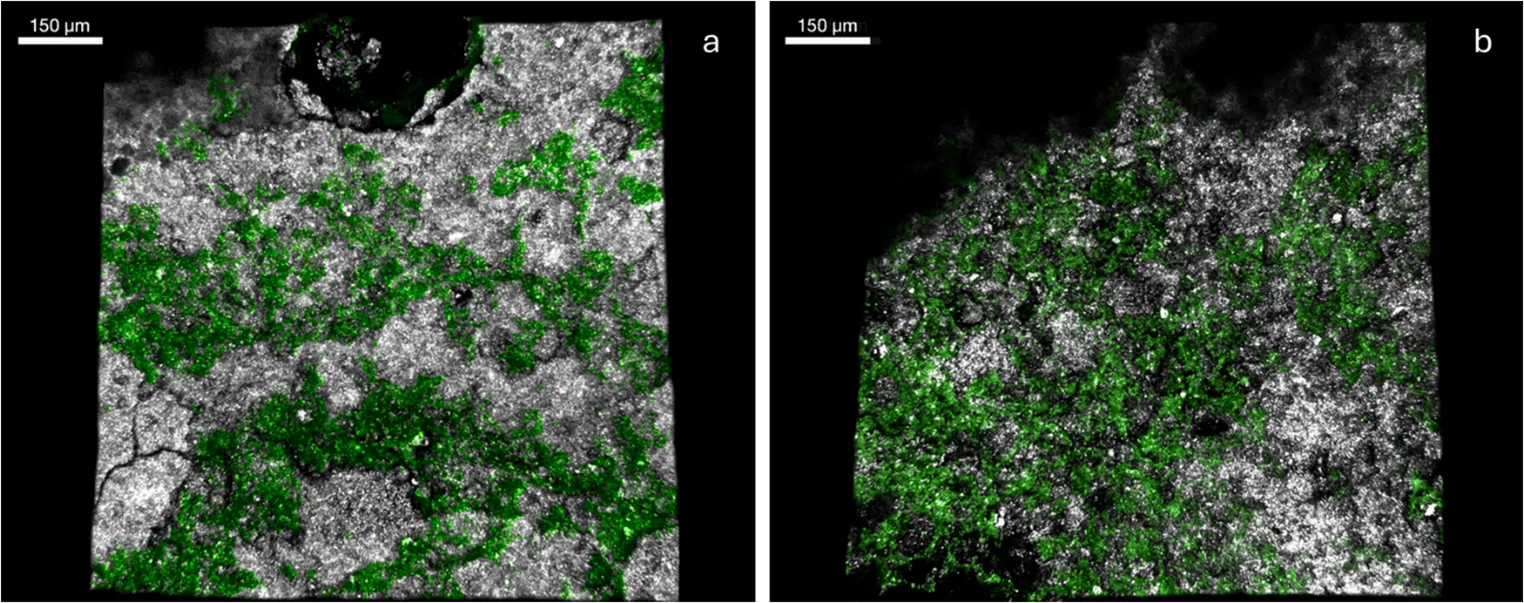
Confocal laser scanning microscopy (CLSM) images (20× objective) of biofilms collected from the north (**a**) and south (**b**) walls. Green fluorescence (SYTO9) indicates total biomass, while red fluorescence corresponds to the autofluorescence of photosynthetic microorganisms. Grey signal represents reflectance mode imaging of the stone substrate. Scale bar = 150 μm

CLSM analysis confirmed the absence of photosynthetic microorganisms, as no red autofluorescence was detected (Fig. 5). Total biofilm biomass did not differ significantly between the north (4.17 ± 0.32 μm³/µm²) and south (4.25 ± 0.55 μm³/µm²) walls (*p* > 0.05).

**Fig. 6:**
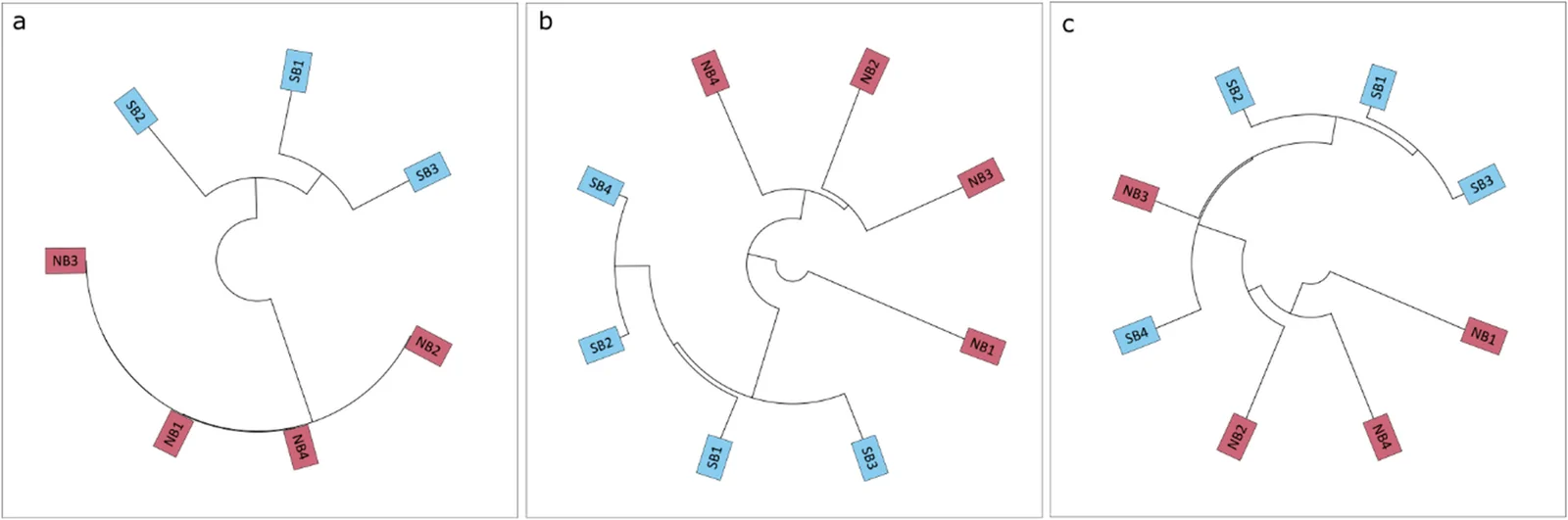
Hierarchical clustering based on Bray–Curtis dissimilarity of archaeal (**a**), bacterial (**b**) and fungal (**c**) communities in biofilms from the north (NB) and south (SB) walls

**Fig. 7:**
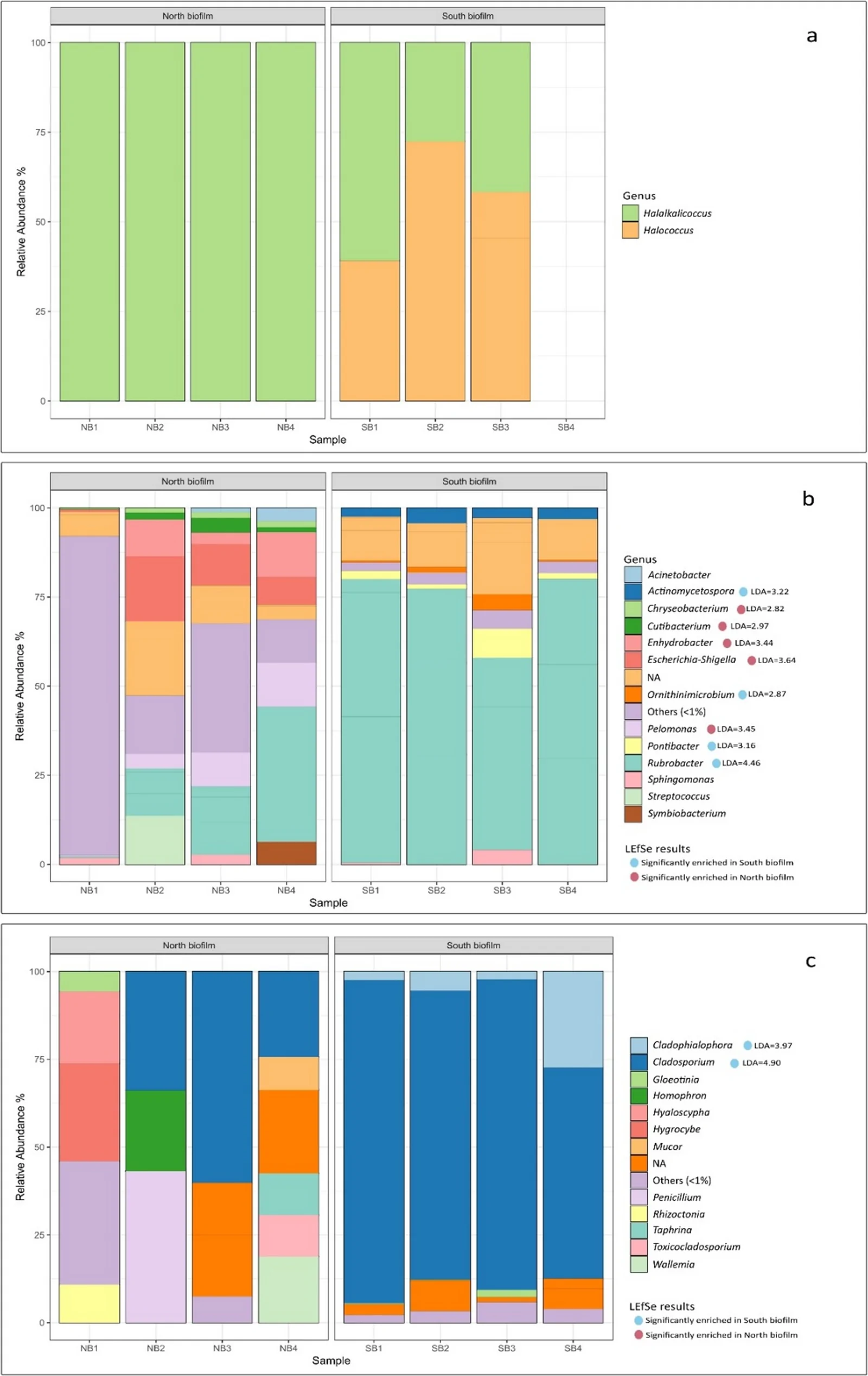
Barplots showing the relative abundance of archaeal (**a**), bacterial (**b**), and fungal (**c**) communities in biofilm samples from the north and south walls, at the genus level. LEfSe results are overlaid in the legend: colored dots mark genera significantly enriched in North (pink) or South (blue) biofilms, and LDA scores indicate the effect size (discriminatory power)

Taxonomic profiles revealed clear compositional differences between the two pink biofilms (Fig. 6). Bray-Curtis clustering showed a clear separation of the archaeal communities (Fig. 6a). Only two archaeal genera were detected (Fig. 7a): *Halalkalicoccus* dominated the north wall (close to 100%), whereas *Halococcus* occurred exclusively in the south (~ 50% relative abundance) (Fig. 7a). No archaeal biomarkers were identified, likely due to low diversity. At the phylum level, all Archaea belonged to *Halobacterota* (100% at both sites).

Bacterial communities also separated clearly between walls (Fig. 6b). The south biofilm was dominated by the genus *Rubrobacter* (~ 73%; ~18% in north), identified as a south biomarker (LDA = 4.47, *p* = 0.0209). Additional south-associated biomarkers included *Actinomycetospora* (~ 3%; LDA = 3.22, *p* = 0.0139), *Pontibacter* (~ 3%; LDA = 3.17, *p* = 0.0139), and *Ornithinimicrobium* (LDA = 2.87, *p* = 0.0139). Conversely, the north biofilm (Fig. 7b) showed higher relative abundances of *Cutibacterium* (north = ~ 2.2%, south = ~ 0.005%), *Escherichia-Shigella* (north = ~ 10.5%, south = ~ 0.03%), and *Pelomonas* (north = ~ 7.7%, south = ~ 0.02%), also identified by LEfSe as biomarker for the north biofilm (*Cutibacterium*: LDA = 2.98, *p* = 0.0139; *Escherichia–Shigella*: LDA = 3.64, *p* = 0.0180; *Pelomonas*: LDA = 3.46, *p* = 0.0139). LEfSe additionally highlighted *Chryseobacterium* (LDA = 2.83, *p* = 0.0139) and *Enhydrobacter* (LDA = 3.45, *p* = 0.0472) as north-associated biomarkers. At the phylum level, the south biofilm was dominated by *Actinomycetota* (~ 93.9%), whereas the north showed a more even distribution between *Actinomycetota* (~ 34.5%) and *Pseudomonadota* (~ 36.4%), with minor *Bacillota* (~ 7.2%) and *Bacteroidota* (~ 3.1%).

Fungal profiles also differed between the two biofilms (Fig. 6c). The south biofilm was dominated by *Cladosporium* (~ 80%; 29% in north) (Fig. 7c), identified as a south biomarker (LDA = 4.41, *p* = 0.0433), together with *Cladophialophora* (LDA = 3.65, *p* = 0.0180). No fungal biomarkers were detected for the north site. At the phylum level, fungi were mainly Ascomycota at both sites but more abundant in the south (~ 98.7% vs. ~ 70.8% in north); Basidiomycota were reduced in the south (~ 1.2%) and remained substantial in the north (~ 26.9%), while minor Mucoromycota occurred only in the north (~ 2.35%).

## Discussion

Chemical analysis revealed marked differences between the two walls (pH, Mg, Al, P, Ca, Fe, TN, TC; Table S3; Fig. 2), despite similar mineralogy (quartz, calcite, plagioclase, K-feldspar, clay minerals, and micas; Table S4). In this alpine environment, seasonal temperature–moisture fluctuations likely drove secondary processes (e.g., salt crystallisation) that generated these contrasts, with salts likely originating from environmental deposition [24]. Higher TC and TN contents observed on the south wall (Table S3) may indicate a larger pool of C- and N-bearing substrates potentially available for microbial colonisation [25, 26]. The chemical divergence was further supported by the north wall’s higher pH than the south wall (Table S3). The salt efflorescence identified by XRD as MgCO_3_ and MgSO_4_ (Table S5) is often found on cultural heritage and is associated with mechanical stress through repeated crystallisation–hydration cycles on wall substrates [27, 28]. In alpine conditions (freeze–thaw and variable moisture), these cycles may have been intensified [2, 3]. Overall, the resulting chemical divergence likely created distinct microhabitats, consistent with the visible differences in rosy discolouration: Raman detected bacterioruberin only in the north biofilm [21, 22] (Fig. 3) and colourimetry confirmed strong chromatic separation between the two biofilms (Table 1). Accordingly, bacterial, archaeal, and fungal beta-diversities were clearly separated by wall orientation (Fig. 6), supporting the idea that small-scale chemical variability, and more broadly substrate properties, drive microbial community assembly in subaerial biofilms [29, 30].

Microscopic investigations revealed the absence of photosynthetic microorganisms at both wall sites (Fig. 5). CLSM has proven particularly valuable for imaging cyanobacteria and algae [31, 32], therefore, the lack of detectable autofluorescent signals in the samples allowed us to exclude a contribution from photosynthetic microorganisms to rosy discolouration.

We examined archaeal, bacterial, and fungal genus-level biomarkers associated with rosy discolouration. No archaeal biomarkers were identified, likely due to the extremely low diversity (only two taxa). *Halalkalicoccus* was detected in both biofilms, whereas *Halococcus* occurred only in the south biofilm (Fig. 7a). Both genera are carotenoid (bacterioruberin) producers and have been reported in pink biofilms on cultural heritage [7, 21].

Four bacterial biomarkers were detected on the south wall (Fig. 7b). *Ornithinimicrobium* and *Actinomycetospora* are not commonly reported but have been detected on cultural heritage and built surfaces [33, 34]. The other two biomarkers, *Rubrobacter* and *Pontibacter*, are carotenoid producers frequently reported on cultural heritage materials [21, 35] and are often linked to pink biofilms [9, 11, 21]. However, in the south, the rosy discolouration was faint, and no pigment was detected by Raman, probably indicating low pigment production. Interestingly, total biofilm biomass quantified by CLSM was comparable between the north and south walls, suggesting that the observed differences in pink hue were likely due to variations in pigment production rather than differences in the abundance of pigmented cells. These taxa were likely selected for their occurrence on oligotrophic, stress-prone geomaterials [36, 37], with *Pontibacter* also including cold-tolerant representatives consistent with alpine conditions [38]. In this context, carotenoids may have functioned primarily as protective compounds against desiccation, oxidative stress, and low temperatures [36, 39], rather than being primarily responsible for the intense pink colour [9, 36]. This suggests that the observed rosy discolouration followed a pattern different from that typically reported in the literature, where pink biofilms are most often associated with salt efflorescence [7, 8, 21].

By contrast, in the north biofilm, five biomarkers were found. Among them, *Cutibacterium*,* Escherichia–Shigella*, and *Enhydrobacter* (Fig. 7b) are typical human-/indoor-associated Bacteria and are often detected on publicly exposed cultural heritage [40–42], while *Pelomonas* is more commonly linked to oligotrophic freshwater and engineered water systems [43]. Finally, *Chryseobacterium* (Fig. 7b) is less frequently observed among the taxa typically associated with pink biofilms and, to our knowledge, has not been directly linked to rosy discolouration on monuments. This genus includes halotolerant and cold-tolerant species [44, 45], consistent with its ability to persist under elevated salinity and alpine environments, which may explain its biomarker status. Notably, *Chryseobacterium* strains produce flexirubin-type pigments that shift from yellow-orange to pink under strongly alkaline conditions [46]. This was consistent with the strong rosy discolouration and the strongly alkaline pH (Table S2) in the north wall. However, no clear flexirubin signature was detected by Raman, likely because published reference Raman spectra for flexirubin are scarce and not derived from *Chryseobacterium* [47], and its concentration may have been below the instrumental detection limit. In fact, as observed on the south wall, pigments could still be present at low abundance, sufficient to produce a measurable colour shift (also supported by colourimetry), yet remaining undetectable by Raman. This interpretation therefore remains tentative, as pigment identity was not confirmed by direct extraction or chromatographic analyses (e.g., HPLC). In contrast, Raman revealed a clear bacterioruberin signal. The detection of taxa reported to produce bacterioruberin-type carotenoids, such as *Rubrobacter* and *Halalkalicoccus*, although with lower abundance, further supported the potential presence of this pigment [9, 48]. Bacterioruberin is a hallmark pigment of salt-adapted lifestyles; it stabilises membranes, reduces water and ion loss, and protects against oxidative stress, low temperatures, and UV radiation [9, 10, 49]. Thus, the pink biofilm from the north wall likely reflected contributions from both flexirubin-type pigments and bacterioruberin.

In the fungal communities, two biomarkers were found for the south biofilm: *Cladosporium* and *Cladophialophora*. *Cladosporium* is consistent with its well-documented ubiquity on building and stone surfaces [35]. *Cladophialophora* (Fig. 7c) is a melanised genus reported on cultural heritage and mineral surfaces in oligotrophic, subaerial environments, tolerant to temperature and hydration fluctuations [50], which was in line with its presence at our site. Conversely, the absence of north fungal biomarkers (Fig. 7c) suggested that no single fungal taxon was selectively favoured on the north wall.

## Conclusions

The archaeal, bacterial, and fungal communities show both consistency with and divergence from patterns reported in the literature. Particularly, *Halalkalicoccus* and *Halococcus*, are among the most frequently reported halophilic Archaea associated with pink biofilms on cultural heritage surfaces [7, 21]. Similarly, bacterial genera such as *Rubrobacter* and *Pontibacter* have been repeatedly implicated in rosy discolouration across diverse environments [9, 21, 35]. However, the identification of *Chryseobacterium* as a potential biomarker highlights a less commonly reported taxon, suggesting that additional microbial contributors may be involved, particularly under specific environmental conditions. In contrast, the fungal communities appeared less specific, with genera such as *Cladosporium* and *Cladophialophora* reflecting their widespread occurrence on stone substrates rather than a direct or exclusive association with pink pigmentation [35].

Overall, our results suggested that wall-specific microhabitats shaped both microbial biomarkers and pigment expression underlying rosy discolouration. By identifying microbial biomarkers, including the potential role of *Chryseobacterium* and its flexirubin-type pigments, our results expanded the range of taxa and pigments associated with pink biofilms, addressing which microbes contributed to rosy discolouration at higher altitudes. Pigment assignments were based on spectroscopic evidence; direct extraction and chromatographic confirmation were not feasible due to limited sample availability and conservation constraints, representing a methodological limitation. The findings also clarify the relationship with salt efflorescence: pink biofilm can establish even in the absence of salts, as on the south wall, suggesting selection by other environmental stressors. However, salt-rich and alkaline microhabitats may enhance pigment expression, amplifying discolouration where efflorescence occurs. Overall, these results support our hypothesis that salt acts as an amplifying rather than essential factor in rosy discolouration. These findings highlighted that colour intensity does not directly reflect microbial biomass and should therefore be interpreted cautiously in heritage diagnostics. More broadly, they supported an ecological framework to guide integrated monitoring and preventive conservation in high-altitude heritage sites, addressing both crystallisation-driven damage and discolourations.

## Supplementary Information

Below is the link to the electronic supplementary material.

Supplementary Material 1 (DOCX 75.1 KB)

## Data Availability

The sequencing data generated in this study are available in the NCBI Sequence Read Archive (SRA) under accession number PRJNA1438727. Fully reproducible code associated with the bioinformatic analyses is publicly available in the GitHub repository: https://github.com/SoilMolecularEcology/metabarcoding-workflows/tree/main/articles/rosy_discolouration-alpine-chapel.
