# Supplementary material for: Rosy Discolouration in an Alpine Chapel: Beyond Salt Dependence
Source: Microb Ecol. 2026 May 27;89:151. doi: 10.1007/s00248-026-02795-2 (PMC13402261; doi:10.1007/s00248-026-02795-2)
Supplement: Supplementary file 1 [file 248_2026_2795_MOESM1_ESM.docx]

**Supplementary Material**

**Rosy Discolouration in an Alpine Chapel: Beyond Salt Dependence**

Marzanni, Alessia^1,2 *^, Landolfi, Maria^1^, Tiziani, Raphael^1,3^, Bombardelli, Sabrina ^4^, Celi, Domenico^4^, Pittertschatscher, Martin^5^, Buttarelli, Alessia^6^, Bruni, Silvia^6^, Pecchioni, Elena^7^, Perito, Brunella^4^, Cnudde, Veerle^2,8^, Cappitelli, Francesca^9^, Mimmo, Tanja^1, 3^, Villa, Federica^9^, Borruso, Luigimaria^1, 3 *^

^1^ Faculty of Agricultural, Environmental and Food Sciences, Free University of Bozen-Bolzano, 39100 Bolzano, Italy

^2^ PProGRess-UGCT, Department of Geology, Ghent University, Ghent, Belgium

^3^ Competence Centre for Plant Health, Free University of Bozen-Bolzano, Bolzano, Italy

^4^ Department of Biology, Università degli Studi di Firenze, Florence, Italy

^5^ Associazione Restauratori-Conservatori Alto Adige (ARCA), Italy

^6^ Department of Chemistry, Università degli Studi di Milano, Milan, Italy

^7^ Department of Earth Sciences, Università degli Studi di Firenze, Florence, Italy

^8^ Environmental Hydrogeology, Department of Earth Sciences, Utrecht University, Utrecht, the Netherlands

^9^ Department of Food, Environmental and Nutritional Sciences, Università degli Studi di Milano, Milan, Italy

*Corresponding authors: Alessia Marzanni ([Alessia.Marzanni@student.unibz.it](mailto:Alessia.Marzanni@student.unibz.it)) and Luigimaria Borruso ([luigimaria.borruso@unibz.it](mailto:luigimaria.borruso@unibz.it)).

**Supplementary tables**

**Supplementary Table 1**. Overview of the analyses performed on each sampling area illustrated in Fig. 1. Sampling sites include surfaces with and without biofilms on both the north and south walls, as well as an area affected by salt efflorescence. Chemical analysis includes ICP-MS, TC, TN and pH.

| **Number ID in Fig. 1** | **Type of sample** | **Colorimetric analysis-**in situ | **Chemical analysis** | **Raman spectroscopy and spectrofluorimetric** | **Confocal Microscopy** | **Genomic**  **analysis** | **XRD analysis** |
| --- | --- | --- | --- | --- | --- | --- | --- |
| 1 | North wall with biofilm | 5 measures | 4 replicas | 1 replica | 5 measures | 4 replicas | 3 replicas |
| 2 | North wall | 5 measures | 2 replicas | 1 replica | - | - | - |
| 3 | Salt efflorescence | - | 1 replica | - | - | - | 3 replicas |
| 4 | South wall with biofilm | 5 measures | 4 replicas | 1 replica | 5 measures | 4 replicas | 3 replicas |
| 5 | South wall | 5 measures | 2 replicas | 1 replica | - | - | - |

**Supplementary Table 2.** Summary of sequencing output after quality-control (QC) filtering for bacterial, archaeal, and fungal datasets, including total reads retained, average reads per sample, total number of OTUs, and number of samples analysed. Values refer to the non-rarefied datasets.

|  | **Reads after**  **QC filtering** | **Average**  **reads** | **Total number**  **of OTU** | **Number of**  **samples** |
| --- | --- | --- | --- | --- |
| **Bacteria** | 1021143 | 127643 | 817 | 8 |
| **Archaea** | 1219680 | 174240 | 8 | 7 |
| **Fungi** | 1745556 | 218195 | 54 | 8 |

**Supplementary Table 3.** Chemical parameters of the north wall with and without biofilm, the south wall with and without biofilm, and the salt efflorescence from the north wall. Mean concentrations, standard deviations, and p-values are reported Means were compared using a one-way ANOVA or the Kruskal-Wallis test when the assumption of normality was not met. Statistically significant p-values are shown in bold (p < 0.05). Letters (a,b,c) indicate the statistical class, n.s. = not significant. TC: total carbon; TN: total nitrogen; TC/TN: total carbon to total nitrogen ratio.

|  | **North Wall Biofilm** | **North Wall** | **South Wall**  **Biofilm** | **South Wall** | **Salt  efflorescence** | **p-value** |
| --- | --- | --- | --- | --- | --- | --- |
| **Na (mg g^-1^)** | 6.20 ± 1.88^a^ | 1.21 ± 0.005^ab^ | 0.94 ± 0.04^ab^ | 0.66 ± 0.05^b^ | 113.6 | **0.0173** |
| **Mg (mg g^-1^)** | 10.9 ± 0.63^a^ | 14.8 ± 7.98^ab^ | 78.0 ± 0.87^b^ | 47.9 ± 33.6^ab^ | 9.84 | **0.0263** |
| **Al (mg g^-1^)** | 7.69 ± 0.66^ab^ | 8.85 ± 0.61^a^ | 6.83 ± 0.84^ab^ | 4.95 ± 2.28^b^ | 4.19 | **0.0305** |
| **P (mg g^-1^)** | 0.24 ± 0.01^c^ | 0.26 ± 0.003^bc^ | 0.33 ± 0.03^ab^ | 0.37 ± 0.03^a^ | 0.13 | **0.0018** |
| **S (mg g^-1^)** | 1.63 ± 3.16^a^ | 0.03 ± 0.0005^a^ | 1.08 ± 0.61^a^ | 7.40 ± 3.82^a^ | 82.81 | 0.1244 |
| **K (mg g^-1^)** | 7.52 ± 4.30^a^ | 3.72 ± 0.18^ab^ | 2.04 ± 0.33^ab^ | 1.25 ± 0.48^b^ | 21.00 | **0.0173** |
| **Ca (mg g^-1^)** | 72.9 ± 8.12^b^ | 47.5 ± 11.7^b^ | 80.3 ± 10.0^ab^ | 114 ± 26.7^a^ | 21.66 | **0.0058** |
| **Ti (mg g^-1^)** | 0.22 ± 0.03^a^ | 0.30 ± 0.04^a^ | 0.21 ± 0.02^a^ | 0.21 ± 0.09^a^ | 0.18 | 0.1584 |
| **Mn (mg g^-1^)** | 0.17 ± 0.007^a^ | 0.21 ± 0.03^a^ | 0.17 ± 0.003^a^ | 0.13 ± 0.04^a^ | 0.09 | 0.0725 |
| **Fe (mg g^-1^)** | 10.7 ±0.41^ab^ | 12.5 ± 1.23^a^ | 8.60 ± 0.66^ab^ | 6.37 ± 4.24^b^ | 5.79 | **0.0193** |
| **Cu (mg g^-1^)** | 0.01 ± 0.002^a^ | 0.008± 0.0002^a^ | 0.01 ± 0.001^a^ | 0.03 ± 0.02^a^ | 0.01 | 0.0700 |
| **Zn (mg g^-1^)** | 0.02 ± 0.002^a^ | 0.03 ± 0.001^ab^ | 1.00 ± 0.28^b^ | 0.24 ± 0.16^ab^ | 0.02 | **0.0173** |
| **Sc (mg g^-1^)** | 0.003 ± 0.001^a^ | 0.002 ± 0.0002^a^ | 0.001 ± 0.0003^a^ | 0.001 ± 0.0008^a^ | 0.001 | **0.0390** |
| **Se (mg g^-1^)** | 0.001 ± 0.001^a^ | 0.0007 ± 0.0002^a^ | 0.0008 ± 0.0003^a^ | 0.001 ± 0.0001^a^ | 0.001 | 0.4448 |
| **Mo (mg g^-1^)** | 0.009 ± 0.01^a^ | 0.001 ± 0.001^a^ | 0.0009 ± 0.0002^a^ | 0.003 ± 0.0006^a^ | 0.004 | 0.0776 |
| **Pb (mg g^-1^)** | 0.01 ± 0.002^a^ | 0.007 ± 0.001^a^ | 0.48 ± 0.14^a^ | 0.35 ± 0.31^a^ | 0.00 | **0.0325** |
| **pH** | 10.54 ± 0.18^a^ | 10.25 ± 0.16^a^ | 9.86 ± 0.05^b^ | 9.47 ± 0.01^c^ | 10.25 | **6.70E-05** |
| **TN (%)** | 0.15 ± 0.09^b^ | 0.03 ± 0.01^b^ | 0.23 ± 0.06^ab^ | 0.36 ± 0.11^a^ | 0.11 | **0.0113** |
| **TC (%)** | 4.35 ± 0.52^b^ | 2.80 ± 0.06^b^ | 9.88 ± 1.25^a^ | 8.74 ± 2.37^a^ | 2.33 | **0.0002** |
| **TC/TN** | 35.71 ± 12.89^n.s.^ | 106.14 ± 26.81^n.s.^ | 44.64 ± 5.47^n.s.^ | 24.03 ± 0.58^n.s.^ | 20.67 | 0.0519 |

**Supplementary Table 4.** Qualitative mineralogical composition (XRPD) of three samples from both the north and south walls, with a partial estimate of the minerals' amounts based only on peak elongation. xxx = high amount; xx = medium amount; x = low amount; tr = traces.

|  | **Quartz** | **Calcite** | **Plagioclase/**  **Albite** | **KFeldspar/ Microcline** | **Miche** | **Clay minerals** |
| --- | --- | --- | --- | --- | --- | --- |
| **North wall 1** | xxx | xx | x | tr | xx | x |
| **North wall 2** | xxx | xx | xx | x | xx | x |
| **North wall 3** | xxx | x | x | xx | xx | x |
| **South wall 1** | xxx | xx | xx | x | xx | xx |
| **South wall 2** | xxx | xx | x | x | xx | xx |
| **South wall 3** | xxx | tr | x | x | tr | x |

**Supplementary Table 5.** Qualitative mineralogical composition (XRPD) of three salt efflorescence samples from the north wall, with a partial estimate of the minerals' amounts based only on peak elongation. xxx = high amount; xx = medium amount; x = low amount; tr = traces.

|  | **Nesquehonite** | **Dypingite** | **Hydromagnesite** | **Epsomite** |
| --- | --- | --- | --- | --- |
| **Salt efflorescence 1** | xx | x | xx | x |
| **Salt efflorescence 2** | x | x | x | x |
| **Salt efflorescence 3** | x | x | x | x |

**Supplementary figures**

**Supplementary Figure 1.** Fluorescence emission spectra (λ_exc_ = 532 nm) of the south wall with biofilm (a), the south wall without biofilm (b), the north wall without biofilm (c), and the north wall with biofilm (d)

**Supplementary methods - Bioinformatic workflow**

**##16S rRNA bacterial dataset##**

**##Sequence denoising, quality filtering, chimera removal, and ASV inference using DADA2##**

*qiime dada2 denoise-paired \
 --i-demultiplexed-seqs demux-paired-end_16S_bac.qza \
 --p-trim-left-f 21 \
 --p-trim-left-r 21 \
 --p-trunc-len-f 235 \
 --p-trunc-len-r 245 \
 --o-table table_16S_bac.qza \
 --o-representative-sequences rep-seqs_16S_bac.qza \
 --o-denoising-stats denoising-stats_16S_bac.qza \
 --p-n-reads-learn 500000 \
 --verbose \
 --p-max-ee-f 2 \
 --p-max-ee-r 2*

**##De novo clustering at 97% sequence identity using VSEARCH##**

*qiime vsearch cluster-features-de-novo \*
 *--i-table table_16S_bac.qza \
 --i-sequences rep-seqs_16S_bac.qza \
 --p-perc-identity 0.97 \
 --o-clustered-table table-16S-dn-97_bac.qza \
 --o-clustered-sequences rep-seqs-16S-dn-97_bac.qza*

**##Taxonomic assignment using the SILVA v138-99 classifier##**

*qiime feature-classifier classify-sklearn \
 --i-classifier silva-v138-99-nb-classifier.qza \
 --i-reads rep-seqs-16S-dn-97_bac.qza \
 --o-classification taxonomy-dn-16S-97_bac.qza \
 --verbose \
 --p-n-jobs -2 \
 --p-reads-per-batch 100000*

**##16S rRNA archaeal dataset##**

**##Sequence denoising, quality filtering, chimera removal, and ASV inference using DADA2##**

*qiime dada2 denoise-paired \
 --i-demultiplexed-seqs demux-paired-end_16S_arch.qza \
 --p-trim-left-f 21 \
 --p-trim-left-r 21 \
 --p-trunc-len-f 235 \
 --p-trunc-len-r 245 \
 --o-table table_16S_arch.qza \
 --o-representative-sequences rep-seqs_16S_arch.qza \
 --o-denoising-stats denoising-stats_16S_arch.qza \
 --p-n-reads-learn 50000 \
 --verbose \
 --p-max-ee-f 2 \
 --p-max-ee-r 2*

**##De novo clustering at 97% sequence identity using VSEARCH#**

*qiime vsearch cluster-features-de-novo \
 --i-table table_16S_arch.qza \
 --i-sequences rep-seqs_16S_arch.qza \
 --p-perc-identity 0.97 \
 --o-clustered-table table-16S-dn-97_arch.qza \
 --o-clustered-sequences rep-seqs-16S-dn-97_arch.qza*

**##Taxonomic assignment using the SILVA v138-99 classifier##**

*qiime feature-classifier classify-sklearn \
 --i-classifier silva-v138-99-nb-classifier.qza \
 --i-reads rep-seqs-16S-dn-97_arch.qza \
 --o-classification taxonomy-dn-16S-97_arch.qza \
 --verbose \
 --p-n-jobs -2 \
 --p-reads-per-batch 100000*

**##ITS fungal dataset##**

**##Sequence denoising, quality filtering, chimera removal, and ASV inference using DADA2##**

*qiime dada2 denoise-paired \
 --i-demultiplexed-seqs demux-paired-end_ITS.qza \
 --p-trim-left-f 21 \
 --p-trim-left-r 21 \
 --p-trunc-len-f 210 \
 --p-trunc-len-r 250 \
 --o-table table_ITS.qza \
 --o-representative-sequences rep-seqs_ITS.qza \
 --o-denoising-stats denoising-stats_ITS.qza \
 --p-n-reads-learn 500000 \
 --verbose \
 --p-max-ee-f 2 \
 --p-max-ee-r 2*

**##De novo clustering at 97% sequence identity using VSEARCH##**

*qiime vsearch cluster-features-de-novo \
 --i-table table_ITS.qza \
 --i-sequences rep-seqs_ITS.qza \
 --p-perc-identity 0.97 \
 --o-clustered-table table-ITS-dn-97.qza \
 --o-clustered-sequences rep-seqs-ITS-dn-97.qza*

**###Taxonomic assignment using the UNITE classifier##**

*qiime feature-classifier classify-sklearn \
 --i-classifier unite-INSD-v10.0-classifier.qza \
 --i-reads rep-seqs-ITS-dn-97.qza \
 --o-classification taxonomy-dn-ITS-97.qza \
 --verbose \
 --p-n-jobs -2 \
 --p-reads-per-batch 100000*
